# A Comparison of Surgical Invasions for Spinal Nerve Ligation with or without Paraspinal Muscle Removal in a Rat Neuropathic Pain Model

**DOI:** 10.1155/2016/6741295

**Published:** 2016-08-11

**Authors:** Yi-Gang Huang, Qing Zhang, Hao Wu, Chang-Qing Zhang

**Affiliations:** ^1^Department of Orthopedics, Shanghai Sixth People's Hospital, Shanghai Jiao Tong University, 600 Yi Shan Road, Shanghai 200233, China; ^2^Department of Pharmacology, Shanghai Institute of Materia Medica, 555 Zu Chong Zhi Road, Shanghai 201203, China

## Abstract

L5 spinal nerve ligation (SNL) in rats is one of the most popular models for studying neuropathic pain because of its high reproducibility. During the surgery, a part of the L5 paraspinal muscle is usually removed, which produces extra trauma and may potentially affect the physiological processes involved in neuropathic pain. To reduce the surgical trauma, the paraspinal muscle retraction was developed for exposure of the spinal nerve. The current study was aimed at comparing the surgical invasions between the L5 SNL models with paraspinal muscle removal or retraction. The results showed that both methods induced similar neuropathic pain behavior. However, the paraspinal muscle retraction group exhibited an average of 2.7 mg less blood loss than the muscle removal group. This group also showed a significantly lower increase in serum myoglobin and creatine phosphokinase levels on postoperative days 1 and 2, as well as a lower increase in interleukin-1*β* and interleukin-6 levels on postoperative day 1. The paraspinal muscle maintained normal morphological features following paraspinal muscle retraction. Our results indicate that the SNL rat model with paraspinal muscle retraction is a reliable physiological model that is reproducible, readily available, and less invasive than the model with muscle removal.

## 1. Introduction

Neuropathic pain is a pathophysiological condition that manifests as spontaneous burning pain, abnormal sensitization (hyperalgesia), pain produced by innocuous stimulation (allodynia), and several other sensory symptoms after nerve injury [1]. The prevalence of chronic pain is 25–48% in the general population, and approximately one-fifth of cases of reported chronic pain are thought to be neuropathic in origin [2, 3]. However, the physiological mechanisms that contribute to neuropathic pain have not been fully explored. A number of animal models of peripheral nerve injury have been used as important tools for such research because they induce specific features that are similar to signs of human neuropathic pain, such as hyperalgesia, allodynia, and spontaneous pain. The spinal nerve ligation (SNL) model, which was described by Kim and Chung in 1992 [4, 5], is one of the most widely used models because of its low experimental variability of spontaneous pain and evoked pain (hyperalgesia, allodynia) and the absence of autotomy [6–10]. To expose the L5 spinal nerve at a point just distal to the dorsal root ganglion (DRG) for ligation, the paraspinal muscles from the level of the L5 spinous process to the sacrum must usually be removed, and then the L6 transverse process is removed. Compared to other neuropathic models, for example, the chronic constriction injury model or the partial sciatic nerve ligation model, SNL requires more extensive surgery. The removal of the paraspinal muscles may produce more muscle damage and further complicate the pathologic mechanisms involved in neuropathic pain. Paraspinal muscle injury is usually considered to play a key role in the development of lower back pain because of the abundant nociceptive fiber innervation of this area [11]. Therefore, paraspinal muscle damage should be carefully controlled to reduce its impact on the physiological processes related to neuropathic pain during the SNL procedure. Alternatively, the paraspinal muscle can be retracted for exposure of the spinal nerve [12, 13]. We also performed the surgery by retracting the L5 paraspinal muscle laterally without the muscle removal. This method may cause less damage to muscles, but reduced visualization may result in a higher risk of neurovascular injury and blood loss. One of the disadvantages of the model is its vulnerability to L4 spinal nerve injury. Even a slight damage to the L4 may eliminate the responses of allodynia [7]. Little is known about the differences in efficiency or in the degree of approach-related trauma in performing the SNL procedure with and without paraspinal muscle removal.

The aim of the present study was to compare the neuropathic behaviors and levels of biochemical markers of muscle damage and inflammation that result from the two procedures of SNL in adult rats. Creatine phosphokinase (CK) and myoglobin (Myo) were measured before and after SNL surgical procedures and used as universal markers for muscle damage. Interleukin-1*β* (IL-1*β*), interleukin-6 (IL-6), and tumor necrosis factor-alpha (TNF-*α*) levels were measured and used as markers for surgery-induced inflammation. Behavioral tests, including mechanical allodynia and thermal hyperalgesia, were used to evaluate neuropathic pain after SNL.

## 2. Materials and Methods

### 2.1. Animals

A total of 40 adult male Sprague-Dawley rats (190–210 g) were randomly divided into 4 groups to undergo (1) L5 SNL with removal of the L5 paraspinal muscle, *n* = 12, (2) sham 1—the sham control with removal of the L5 paraspinal muscle, *n* = 8, (3) L5 SNL with retraction of the L5 paraspinal muscle, *n* = 12, and (4) sham 2—the sham control with retraction of the L5 paraspinal muscle, *n* = 8. The rats were housed under a controlled temperature (22 ± 24°C) and a 12-hour light/dark cycle (lights switched on at 07:00 and off at 19:00). Food pellets and water were supplied ad libitum. All handling and surgical procedures were performed in strict accordance with the guidelines of the IASP [14].

### 2.2. Surgical Procedures

After the rats were intraperitoneally anesthetized with sodium pentobarbital, the left L5 spinal nerve ligation was performed. The line connecting the surface landmarks of the bilateral iliac crests was considered the interspace between the L5 and L6 spinous processes. The surgical procedure was performed under a binocular stereoscopic microscope using 10x magnification. A gelatin sponge was used for intraoperative hemostasis. After sterilization, a longitudinal incision was made 5 mm lateral (left) to the midline from the L5 vertebra to the S1 vertebra. The dorsolumbar fascia, which provides attachment to the latissimus dorsi, was opened, and the left paraspinal muscle was exposed. In the paraspinal muscle removal group, as Kim and Chung [5] described, the paraspinal muscles were isolated using small scissors with blunt tips and then removed from the level of the L5 spinous process to the sacrum. In the paraspinal muscle retraction group, a retractor made in house was used to retract the paraspinal muscle laterally, and then the transverse processes and the lateral surface of the facet joint were exposed. A small rongeur was used to remove the L6 transverse process, which covers the L4 and L5 spinal nerves in a rostral and ventral orientation. In the paraspinal muscle retraction group, a decline of the operating table 20° toward the right was helpful for exposing the left side of the spinal nerve. Once separated from L4 spinal nerve, the L5 spinal nerve was tightly ligated with a piece of 6-0 silk thread and then divided 5 mm distal to the ligation. After hemostasis was confirmed, the wound layers were closed with 4-0 silk thread. The sham control animals were performed identically, with removal or retraction of the L5 paraspinal muscle, except that the nerves were not ligated or sectioned after exposure.

### 2.3. Operative Data

The operative data that were collected included the length of the incision, intraoperative blood loss, and the operative time from incision to closure. The length of the incision was measured with a slide caliper after ligation of the L5 spinal nerve. Blood loss was quantitated by weighing the gelatin sponge before and after operation.

### 2.4. Measurements of Muscle Damage and Inflammation

Blood samples were collected from tail vein for serum separation on preoperative day 1, postoperative days 1, 2, and 7. Serum samples were stored at −80°C until analysis. Serum CK and Myo levels were measured to assess muscle damage. IL-1*β*, IL-6, and TNF-*α* levels were recorded as global measures of inflammation and surgical insult. All of the measurements were acquired using Quantikine ELISA Kits (BD Biosciences, Minneapolis, USA).

### 2.5. Behavioral Tests

All rats were tested for mechanical allodynia and heat hyperalgesia of the plantar surface of the hindpaws 1 day before and 2, 7, and 14 days after surgery.

#### 2.5.1. Mechanical Allodynia

Mechanical allodynia was measured in rats using an electronic von Frey anesthesiometer (Ugo Basile, Italy). During the tests, rats were placed in individual chambers on top of a meshed floor suspended 50 cm above the laboratory bench-top and allowed to adapt to the environment for 30 min prior to each testing session. An electronic von Frey polypropylene tip was applied perpendicularly to the midplantar surface of the hindpaw, and the threshold of the stimulus was automatically recorded when the hindpaw showed a withdrawal response to the mechanical stimulus [15–17]. The paw withdrawal mechanical thresholds were recorded five times at 5-minute intervals, and the mean value of these five consecutive measurements was calculated for each paw.

#### 2.5.2. Heat Hyperalgesia

The rats were placed on a heated (50°C) plate, as described previously [18]. The application of thermal stimuli can cause a rat to withdraw its hindpaws, a behavior that is observed as the licking of the hindpaws. The latency value for hindpaw withdrawal due to the heat stimulus was recorded. For each measurement, the experiment was performed three times at 10 min intervals to obtain a mean latency value.

### 2.6. Histological Tests

After the final behavior test, the rats were sacrificed, and the paraspinal muscles of the L5 segment were bilaterally removed. After the tissues were fixed in formalin for 48 h, the tissues were transversely sectioned and stained with hematoxylin and eosin.

### 2.7. Statistical Analysis

All values were expressed as the mean ± standard deviation. In surgical invasions study, differences in values between the surgical groups and their sham controls were compared using One-Way ANOVA, followed by the Bonferroni test for comparing between the paraspinal muscle retraction and paraspinal muscle removal groups at each time point. In behavioral study, the values were compared between each of the studied groups and its sham operated control using independent samples *t*-test. A difference was accepted as significant if the *P* value < 0.05. The investigator responsible for the quantification and analysis of the results was blind to the experimental status of each animal. Analyses were carried out using SPSS (version 21.0, IBM, Armonk, NY, USA).

## 3. Results

### 3.1. Operative Data

**Figure 1:**
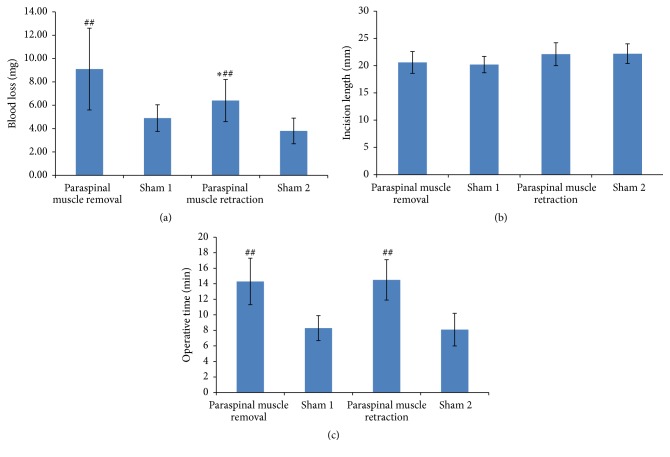
Operative data of the rats. (a) Intraoperative blood loss, (b) incision length, and (c) operative time. ^*∗*^ *P* < 0.05 compared with paraspinal muscle removal. ^##^ *P* < 0.01 compared with the sham operated rats (paraspinal muscle removal group versus sham 1 group, paraspinal muscle retraction group versus sham 2 group).

Operative data were compared between the rats that underwent L5 SNL with removal of the paraspinal muscle and those that underwent L5 SNL with retraction of the paraspinal muscle. As seen in Figure 1, the paraspinal muscle removal group lost an average of 2.7 mg more blood than the paraspinal muscle retraction group, and this difference was significant (9.1 mg versus 6.4 mg, *P* = 0.036). There were no significant differences in the incision length (20.6 mm versus 22.1 mm, *P* = 0.401) or the operative time (14.3 minutes versus 14.5 minutes, *P* = 1.000).

### 3.2. Measurements of Muscle Damage and Inflammation

**Figure 2:**
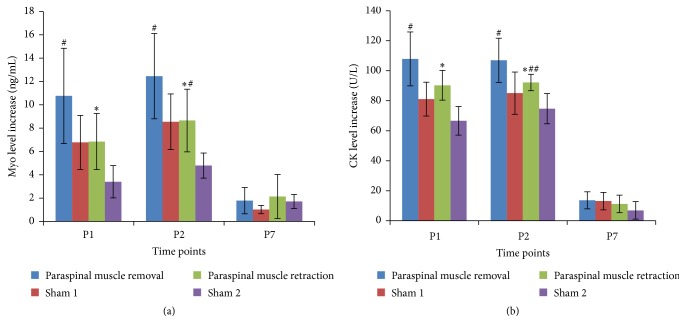
Results showing increased serum levels of serum Myo (a) and CK (b) on postoperative days 1, 2, and 7 after L5 SNL. ^*∗*^ *P* < 0.05 compared with paraspinal muscle removal group. ^#^ *P* < 0.05 compared with the sham operated rats (paraspinal muscle removal group versus sham 1 group, paraspinal muscle retraction group versus sham 2 group). ^##^ *P* < 0.01 compared with the sham operated rats.

The serum levels of Myo and CK greatly increased on postoperative days 1 and 2 and decreased to nearly normal on day 7. The increase was calculated with the serum levels of the markers on each postoperative day minus that on preoperative day 1. The paraspinal muscle retraction group showed a significantly reduced increase in Myo and CK levels on postoperative days 1 and 2 compared to the increase observed in the paraspinal muscle removal group (see Figure 2). On postoperative day 7, the increase in Myo and CK levels was not significantly different between the two groups.

**Figure 3:**
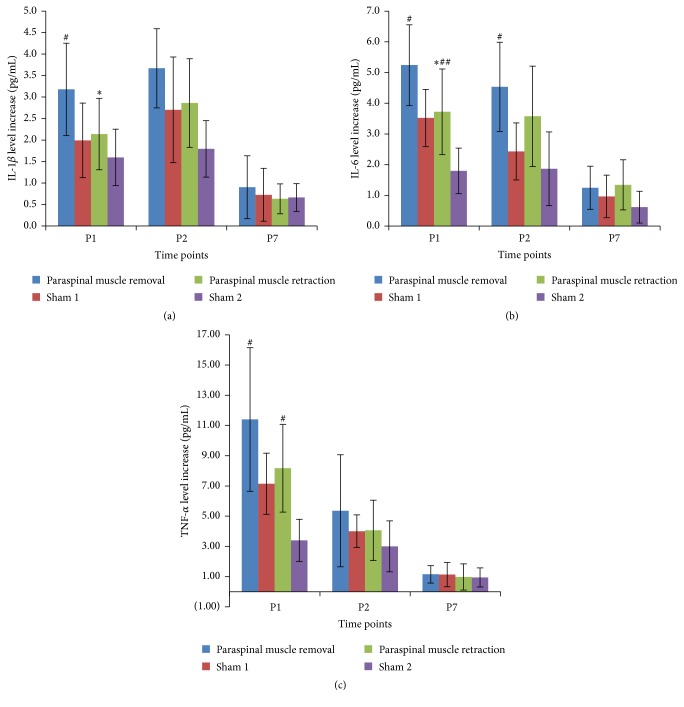
Results show increased serum levels of IL-1*β* (a), IL-6 (b), and TNF-*α* (c) on postoperative days 1, 2, and 7 after L5 SNL. ^*∗*^ *P* < 0.05 compared with paraspinal muscle removal group. ^#^ *P* < 0.05 compared with the sham operated rats. ^##^ *P* < 0.01 compared with the sham operated rats.

There was also a postoperative increase in serum IL-1*β*, IL-6, and TNF-*α* levels in both groups on postoperative days 1 and 2. The paraspinal muscle retraction group showed a significantly reduced increase in IL-1*β* and IL-6 levels on postoperative day 1 compared to the increase observed in the paraspinal muscle removal group (see Figure 3). None of these differences between the two groups reached significance at any other time points. Increase in serum TNF-*α* did not show significant difference between the two groups at any time point.

### 3.3. Behavioral Tests

**Figure 4:**
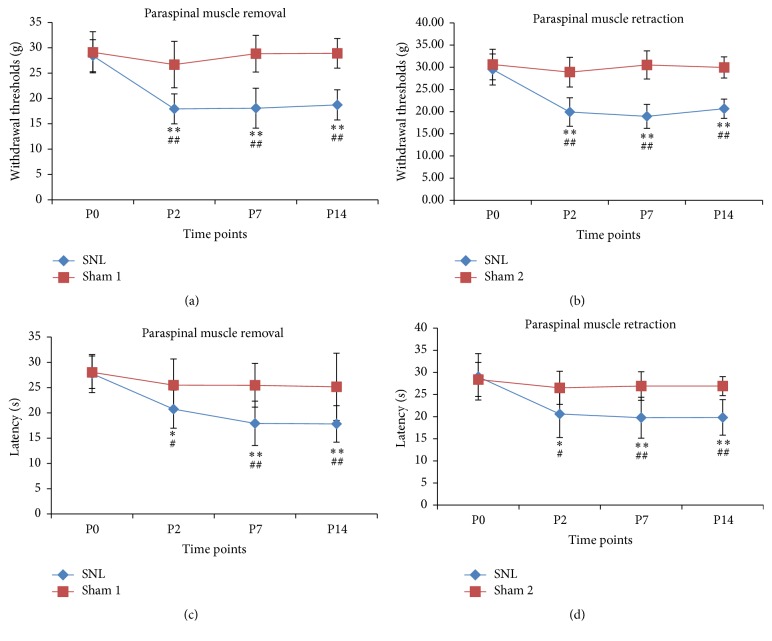
The effects of L5 SNL on mechanical allodynia ((a), (b)) and heat hyperalgesia ((c), (d)) in rats. ^*∗*^ *P* < 0.05 compared with preoperative value. ^*∗∗*^ *P* < 0.01 compared with preoperative value. ^#^ *P* < 0.05 compared with the sham operated rats. ^##^ *P* < 0.01 compared with the sham operated rats.

The hindpaw withdrawal threshold for the rats was greatly decreased on postoperative days 2, 7, and 14. Withdrawal thresholds for rats that underwent paraspinal muscle removal were 28.4 ± 3.1, 17.9 ± 3.0, 18.1 ± 3.9, and 18.7 ± 3.0 g preoperatively and on postoperative days 2, 7, and 14, respectively. For the rats that underwent paraspinal muscle retraction, the withdrawal thresholds were 29.5 ± 3.5, 19.9 ± 3.2, 18.9 ± 2.7, and 20.7 ± 2.2 g at the same time points, respectively. Both groups showed decreased withdrawal thresholds compared with their sham surgery controls at each postoperative time point (see Figures 4(a) and 4(b)).

Similar to the results of mechanical allodynia test, there was a postoperative decrease in the latency of hindpaw withdrawal in the hot plate test. The latency of hindpaw withdrawal was 27.8 ± 3.8, 20.7 ± 3.8, 17.9 ± 4.4, and 19.8 ± 3.6 s preoperatively and on postoperative days 2, 7, and 14, respectively. For the rats that underwent paraspinal muscle retraction, the withdrawal thresholds were 29.0 ± 5.3, 20.6 ± 5.3, 19.7 ± 4.6, and 19.8 ± 4.0 s at the same time points, respectively. Both groups showed decreased latency compared with their sham surgery controls at each postoperative time point (see Figures 4(c) and 4(d)).

### 3.4. Histological Findings

**Figure 5:**
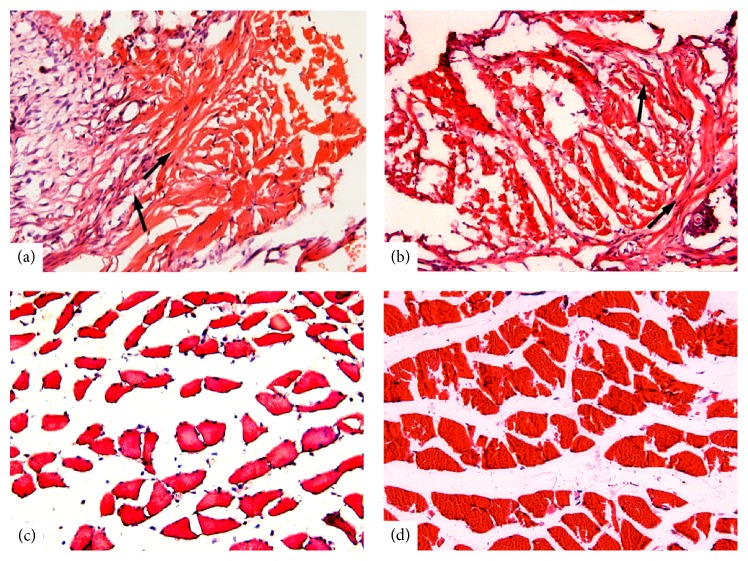
Histological changes in the L5 paraspinal muscle at 2 weeks after L5 SNL and sham surgery. In the rats that underwent L5 SNL (a) or sham (b) surgery with paraspinal muscle removal, the L5 paraspinal muscle was replaced by fibrous connective tissue (arrows). The paraspinal muscle maintained normal morphological features in rats that underwent L5 SNL (c) or sham (d) surgery with paraspinal muscle retraction.

For the rats that underwent paraspinal muscle removal, the removed tissue was replaced by fibrous connective tissue (see Figure 5(a)). In contrast, the paraspinal muscle maintained normal morphological features in rats that underwent paraspinal muscle retraction (see Figure 5(b)).

## 4. Discussion

This study was designed to provide objective measurements of the surgical invasiveness of two approaches to L5 spinal nerve ligation for neuropathic pain. To obtain a true measurement of invasiveness for each procedure, we used three different criteria: operative data (incision length, operative time, and blood loss), muscle damage, and inflammation.

The operative data were used to evaluate whether the lumbar paraspinal muscles hampered the exposure or ligation of the L5 spinal nerve. Inadequate exposure may produce more invasiveness, for example, which can result in a longer incision length, increased operative time, or more blood loss. We observed no difference in these factors between the two groups, which suggests that an experienced operator can complete the surgery smoothly without removing the paraspinal muscles. The lateral retraction of the muscles provided good exposure for the ligation of the L5 spinal nerve. The behavior tests showed that both methods of the SNL model induced significant mechanical allodynia or heat hyperalgesia, while the sham surgery controls did not. The results suggest that the SNL model can be performed successfully without removing the paraspinal muscles.

Serum markers of muscle damage and inflammation have been systematically analyzed in spinal nerve-ligated animals. The true measure of the invasiveness of a procedure is a matter of current debate. We evaluated muscle damage, which is an important aspect of invasiveness. Myo is a low molecular mass cytoplasmic protein that is present mainly in skeletal and heart muscles. Skeletal muscle damage releases Myo into the serum [19]. Due to the high Myo concentration in skeletal muscle tissue, even minor skeletal muscle injuries can result in a significant increase in the Myo concentration in serum. After exertion, Myo can increase within 30 min and remain increased for 5 days [20]. Similarly, CK is a dimeric globular protein that is contained in skeletal myocytes and that produces creatine and ATP from creatine phosphate and adenosine diphosphate. CK flows out of the cell following damage to the cell membrane, which leads to an increase in the concentration of CK in plasma [21, 22]. Therefore, both Myo and CK are useful biochemical markers of muscle damage. In the present study, we measured serum Myo and CK levels to determine the degree of skeletal muscle damage. In both groups, serum CK and Myo levels were greatly increased on postoperative days 1 and 2. However, in the paraspinal muscle removal group, the increases in the CK and Myo levels were much higher than the increases observed in the paraspinal muscle retraction group, despite similar preoperative levels. These results suggest that retraction of the paraspinal muscle, rather than removal, may produce less muscle damage. Further research is needed to determine the relationship between biomarkers and neuropathic pain.

We also analyzed postoperative inflammation to evaluate the invasiveness of the L5 SNL procedures. Tissue damage can trigger an inflammatory response, which can result in an increase in serum levels of proinflammatory cytokines. During the acute phase response, the synthesis and release of proinflammatory cytokines, such as IL-1, IL-6, and TNF-*α*, can increase up to 30-fold [23]. In muscle injury, the cytokine levels play a central role in inflammation and are involved in the repair process. The hemorrhage and edema occur immediately after muscles injury and are accompanied by elevated levels of cytokines [24–26]. Furthermore, changes in cytokine levels are dependent on the degree of injury. An experimental study demonstrated that more severe injury to muscles produced increased cytokine production and a greater degree of inflammation [27]. Clinical studies usually measure the changes of the cytokine levels to evaluate the surgical invasions [28, 29]. Therefore, we used changes in serum levels of these markers to compare the invasiveness of the two approaches for L5 SNL and their controls. Here, we have shown that SNL results in a significant increase in serum cytokine levels on days 1 and 2 after surgery. In the paraspinal muscle removal group, the increases in the IL-1*β* and IL-6 levels were significantly higher than those observed in the paraspinal muscle retraction group on postoperative day 1. The results suggest that preservation of the intact paraspinal muscle during SNL may produce less inflammatory response at early postoperative stage, even though the inflammatory response was also influenced by other procedures, such as ligation of the spinal nerve. Further studies are necessary to work out if serum cytokine levels could clearly influence the presence of neuropathic pain in rats.

The importance of paraspinal muscle preservation lies in how it relates to symptoms of pain because paraspinal muscle injury is potentially implicated in the genesis of back pain. In humans, the paraspinal muscles play essential roles in the stabilization and movement of the spine [30]. The functional and morphological characteristics of the paraspinal muscles are not as well described in laboratory animals, such as rats, as they are in humans [31, 32]. L4–L6 segments of the paraspinal muscle in pigs had been observed to develop rapid atrophy after injury to the L3 segment, which suggests that, in quadrupedal animals, the paraspinal muscle injury is also related to symptom duration in lower back pain [33]. We observed that, after paraspinal muscle removal, the gaps were filled with scar tissue, whereas muscle retraction preserved nearly normal muscle fiber morphology. Clinical study demonstrated that the severity of intraoperative damage to the paraspinal muscle was directly correlated with the postoperative low back pain [34]. The mechanism lies in that dense scar formation in the muscle may have interfered with muscle retraction and may have impaired the normal function of the paraspinal muscles. Although its impact on the behaviors of neuropathic pain is still unclear, excessive damage to the muscle during the surgery should be avoided to decrease the interference to the research of neuropathic pain.

## 5. Conclusion

We have shown that an SNL rat model without paraspinal muscle removal is a suitable physiological model that is reproducible, readily available, and less invasive. This technology offers the potential to study neuropathic pain in a model in which less interference is produced by the surgical procedure. This study also provides a reliable method for comparing invasiveness between different animal models of neuropathic pain.
